# Electrochemotherapy with Mitomycin C Potentiates Apoptosis Death by Inhibiting Autophagy in Squamous Carcinoma Cells

**DOI:** 10.3390/cancers13153867

**Published:** 2021-07-31

**Authors:** Maria Condello, Gloria D’Avack, Rosa Vona, Enrico Pierluigi Spugnini, Licia Scacco, Stefania Meschini

**Affiliations:** 1 National Center for Drug Research and Evaluation, National Institute of Health, 00161 Rome, Italy; maria.condello@iss.it (M.C.); gloria.davack@gmail.com (G.D.); 2 Center for Gender-Specific Medicine, National Institute of Health, 00161 Rome, Italy; rosa.vona@iss.it; 3 Biopulse S.r.l., 80132 Naples, Italy; info@enricospugnini.net; 4 Equivet Roma Hospital, 00134 Rome, Italy; licia.scacco@equivet.it

**Keywords:** electrochemotherapy, mitomycin C, squamous carcinoma cell lines, drug resistance, apoptosis, autophagy, veterinary patients

## Abstract

**Simple Summary:**

Internationally, electrochemotherapy is considered an innovative therapeutic strategy capable of increasing the efficacy of lyophobic drugs in many solid tumors. Our study aimed to verify the potentiating effect of electroporation (EP), in combination with the drug mitomycin C (MMC), in oral and laryngeal cancer cells that exhibited intrinsic drug resistance. Our study showed that EP increased MMC cytotoxicity on both lines compared to single treatment; the mechanism of action was due to EP inhibition of the autophagic process induced by MMC, thus favoring apoptosis. EP plus MMC treatment was also performed on two veterinary patients demonstrating its efficacy, tolerability, and clinical feasibility.

**Abstract:**

We investigated the chemosensitizing effect of electroporation (EP), which, using electrical pulses, permeabilizes cancer cells to drugs. The study involved two human hypopharyngeal and tongue carcinoma cell lines. The surface and intracytoplasmic expression of P-gp were evaluated by flow cytometry, demonstrating that both lines were intrinsically resistant. After establishing the optimal dose of mitomycin C (MMC) to be used, in combination with EP, we showed, by both MTT assay and optical and electron scanning microscopy, the potentiating cytotoxic effect of EP with MMC compared to single treatments. Flow cytometry showed that the cytotoxicity of EP + MMC was due to the induction of apoptosis. In addition to verifying the release of cytochrome C in EP + MMC samples, we performed an expression analysis of caspase-3, caspase-9, Akt, pAkt, HMGB1, LC3I, LC3II, p62, Beclin1, and associated proteins with both apoptotic and autophagic phenomena. Our results were confirmed by two veterinary patients in whom the EP + MMC combination was used to control margins after the resection of corneal squamous carcinoma. In conclusion, we affirmed that the effect for which EP enhances MMC treatment is due to the inhibition of the autophagic process induced by the drug in favor of apoptosis.

## 1. Introduction

Electrochemotherapy (ECT) is a loco-regional and non-invasive therapy that combines the administration of anticancer agents, which have poor membrane permeability, with electrical pulses with adequate characteristics (shape, voltage, frequency) [1]. This innovative medical strategy improves the uptake and efficacy of lipophobic anticancer agents, such as bleomycin, cisplatin, or doxorubicin predominantly by cancer cells [2,3]. The efficacy of ECT, as adjuvant therapy, has been validated in veterinary and human oncology, using different anticancer agents against cutaneous and subcutaneous malignant lesions [4]. Although ECT’s efficacy is well-demonstrated on melanoma, this technique can be applied to different types of tumors, such as bone, liver, breast, head, and neck [5]. This therapy reduces patient morbidity, as ECT allows the use of lower doses of drug concentration, thereby limiting side effects and improving tumor control.

Many in vitro studies have been conducted to understand the cellular modifications and molecular pathways of ECT-induced cell death. Our previous study demonstrated that electroporation increased doxorubicin accumulation and cellular distribution on drug-resistant colon cancer cells [3]. Furthermore, electroporation plus cisplatin increased reactive oxygen species and induced apoptosis on triple-negative breast cancer cells [6]. Alternatively, electroporation in combination with bleomycin, cisplatin, or oxaliplatin induces immunogenic cell death on pancreatic cancer cells [7]. In addition to the ability to deliver the drug more efficiently, the ECT showed no alteration in normal tissue [8]. Furthermore, the electrical characteristics have been extensively studied with the vast data produced in the veterinary field [9,10].

We used human hypopharyngeal squamous cell carcinoma (FaDu) and human tongue squamous cell carcinoma (CAL 27) because oral and larynx cancers are the seventh most prevalent cancer in the world. The chemotherapy protocols used in the clinic are carboplatin, mitomycin C, or cetuximab [11]. We chose the combination of EP and mitomycin C (MMC) since there is limited research in the literature on either in vitro or in vivo models or on human or veterinary models that clarify the cellular mechanisms potentially effective in reducing tumor growth.

Therefore, the aim of this work was threefold: First, we evaluated EP parameters such as the duration and number of the electric pulse, the amplitude of the electric field based on the cell type that can influence the transient permeabilization of the membrane, and the in vitro efficiency of the combined treatment (EP + MMC), in order to reduce tumor cell growth [4,12]. The second objective was to identify the main mechanisms underlying MMC drug resistance (such as the reduction of apoptosis and the induction of autophagy) [13]. The third, was to verify in vivo the effectiveness of the combination in reducing tumor mass in the animal.

## 2. Materials and Methods

### 2.1. Cell Cultures

The human hypopharyngeal carcinoma cell line FaDu (ATCC^®^ HTB-43™) and human tongue carcinoma cell line CAL 27 (ATCC^®^ CRL-2095™) were obtained from the American Type Culture Collection (ATCC). Cells were grown as a monolayer in RPMI 1640 (with L-glutamine), 10% fetal bovine serum (FBS) (Hyclone, Europe Ltd., Cramlington, UK), penicillin (50 U/mL), streptomycin (50 µg/mL), and non-essential amino acids (50 µg/mL) in a humidified atmosphere of 5% CO_2_ in a water-jacketed incubator at 37 °C.

### 2.2. Flow Cytometry Analysis of P-gp Expression

For the determination of cell surface P-gp, FaDu, and CAL 27 cells were incubated for 20 min in phosphate-buffered saline (PBS, Sigma-Aldrich, Saint Louis, MO, USA) supplemented with 1% BSA (Sigma-Aldrich), 10% FBS, and 10% human serum AB in order to saturate possible aspecific sites. They were incubated with primary MAb (MRK16, Kamiya, Thousand Oaks, CA, USA) for 30 min at 4 °C, washed with cold PBS, and then incubated with a goat anti-mouse IgG-fluorescein isothiocyanate conjugated (FITC, Sigma-Aldrich) antibody for 30 min at 4 °C. After washing with PBS, dead cells were excluded from the analysis by adding propidium iodide (PI, Sigma-Aldrich) to the cell suspensions before the acquisition. For the determination of intracellular P-gp, FaDu and CAL 27 cells were fixed with 4% paraformaldehyde solution at room temperature for 20 min. After washing with PBS, cells were permeabilized with 0.5% Triton (Sigma-Aldrich) solution in PBS for 5 min at room temperature. After that, immunolabeling continued as for the surface one.

Samples were analyzed using a BDLSRII flow cytometer equipped with 15 mV, 488 nm air argon ion laser, and a Kimmon HeCd 325 nm laser (Becton, Dickinson and Company, Franklin Lakes, NJ, USA). The fluorescence emissions were collected through a 530 nm bandpass filter for FITC and a 575 nm bandpass filter for PI and acquired in log mode. At least 10,000 events were analyzed. For the quantitative evaluation of the protein expression, the mean fluorescence channel (MFC) was calculated by FACS Diva Software (Becton, Dickinson and Company, Franklin Lakes, NJ, USA).

### 2.3. In Vitro Electroporation 

Trains of biphasic electric pulses were generated with a modified clinical electroporator certified for veterinary use and adapted for laboratory applications (Onkodisruptor^®^-lab, Biopulse Biotech, Naples, Italy). Cells were suspended and placed in electroporation cuvettes and exposed to sequences of eight biphasic pulses lasting 50 + 50 μsec with 300 μsec interpulse, at a voltage of 300 V (3.2 ms total time).

### 2.4. In Vitro Treatments

FaDu and CAL 27 cells were detached from the substratum with a mixture of EDTA and trypsin (0.25%) to obtain a cell suspension (100,000 cells/500 µL culture medium).

For dose–response experiments, each cell suspension was treated with different concentrations of MMC (0–500 µM) for 24, 48, and 72 h (starting from a stock solution 3 mM, Sigma-Aldrich). For other assays, cell suspensions were treated with EP, or drug MMC (5 µM) alone, or EP+MMC for 24 or 48 h.

Per sample, 100,000 cells were transferred to the electroporation cuvette (Bio-Rad Laboratories Inc., PA, USA). Sequential bursts of eight biphasic pulses lasting 50 + 50 µs were applied at a voltage of 300 V/cm. After electroporation, cells were transferred to a plate. Concerning single treatment with MMC, cell suspension in 500 µL was treated with MMC 5 µM, and then seeded in a plate. In combined treatment, after electroporation, cells were treated with MMC (5 µM), and directly transferred in a plate.

As a positive control of apoptosis induction, cells were treated with staurosporine (STS, 1 μM, Sigma-Aldrich) for 24 h [14].

### 2.5. MTT Assay

Methyl-thiazolyl-tetrazolium (MTT, Sigma-Aldrich) assay was employed to assess the drug sensitivities of FaDu and CAL 27 cells. After 24, 48, and 72 h of treatments, samples were processed according to Meschini et al., 2017 [15].

### 2.6. Phase Contrast Microscopy

FaDu and CAL 27 cells, untreated, treated with EP alone or MMC alone, or with EP plus MMC, for 24 h were observed with a phase-contrast microscope (OLYMPUS BX51, Olympus Corporation of the Americas, Center Valley, PA, USA) equipped with a CCD camera (Carl Zeiss, Jena, Germany).

### 2.7. Scanning Electron Microscopy (SEM)

To investigate the morphological modifications induced by combined treatment, observations were performed on samples (untreated and treated cells) get ready according to protocols reported by Condello et al., 2019 [16].

### 2.8. Determination of Apoptotic Cell Death by Annexin V-FITC/PI Staining by Flow Cytometry

An Annexin V-FITC/PI apoptosis detection kit (eBioscence, San Diego, CA, USA) was used on untreated and treated samples to investigate apoptosis [16].

Samples were analyzed using a BDLSRII flow cytometer, as previously described in the P-gp expression paragraph. Percentages of apoptotic, necrotic, and viable cells were calculated using the FACS Diva Software (Becton, Dickinson and Company).

### 2.9. Immunolabeling of Cytochrome C and Nuclear Staining

At the end of the treatments, cells were cultured in plates containing glass coverslips for 24 h. Then, the medium was removed, the cells were washed twice with PBS (pH 7.4) and fixed with 4% paraformaldehyde at room temperature for 20 min. After washing with PBS, cells were permeabilized with 0.5% Triton in PBS for 5 min at room temperature, then washed twice for 2 min with PBS. To saturate the nonspecific binding sites, 3% BSA in PBS was added for 10 min at room temperature. After washing with PBS, incubation with primary antibody (anti-cytochrome c, Santa Cruz Biotechnology, Inc., Dallas, TX, USA) in PBS solution for 30 min at 37 °C followed. After washing in PBS, the secondary antibody was added for 30 min at 37 °C in the dark, and finally, the cell nuclei were labeled with Hoechst 33,342 (10 mg/mL; Molecular Probes) for 10 min at 37 °C in the dark. After mounting in PBS: glycerol solution (1:1), samples were observed using a Nikon fluorescence microscope (TE2000-E MOTORIZED FOCUS), and the images were acquired using MetaMorph software.

### 2.10. Western Blotting

The whole-cell extract was obtained in RIPA buffer in the presence of standard protease and phosphatase inhibitors. The protein content was determined with a protein assay reagent (Bio-Rad Laboratories Inc., PA, USA), using bovine serum albumin as a standard. The equal protein content of total cell lysates (30 µg) was resolved on 10% or 12% SDS-PAGE and electrically transferred to PVDF membranes (Bio-Rad Laboratories, Hercules, CA, USA). Membranes were blocked with TBS-T (20 mM Tris-HCl pH 7.4, 150 mM NaCl, 0.02% Tween-20) containing 5% skimmed milk (Bio-Rad Laboratories, Inc., PA, USA), for 1 h at room temperature, and then incubated overnight at 4 °C with primary antibodies diluted in TBS-T containing 2% milk or 5% BSA. MAb: anti-Caspase-9 (Cell Signaling Technology, Inc., Beverly, MA, USA; # 9508; dil.1:1000); anti p-Akt (Santa Cruz; #sc-293125; dil. 1:1000); anti-BCN1 (Santa Cruz; # sc-48381; dil. 1:1000); pAb anti- LC3 (Novus Biological, #NB100-2220; 1:1000); anti Akt (Santa Cruz; # sc-8312; dil. 1:1000); anti-Caspase-3 (Cell Signaling; # 9664; dil.1:1000); anti-HMGB1 (Sigma-Aldrich; #H9664; dil.1:1000), anti-SQSTM1/p62 (Sigma-Aldrich; #P0067; dil.1:1000) primary antibodies were used. After washing in TBS-T three times, immune-complexes were detected with Horse Radish Peroxidase conjugated species-specific secondary antibodies (Jackson Laboratory, Bar Harbor, ME, USA). Membranes were developed using ECL detection reagents (Millipore Corporation, Billerica, MA, USA). Reactive bands were detected by the ChemiDocMP system (Bio-Rad Laboratories Inc., PA, USA) and signal quantification was performed using IMAGE LAB software (Bio-Rad, Laboratories Inc., Philadelphia, PA, USA). To ensure the presence of equal amounts of protein, the membranes were reprobed with an anti-GAPDH (Santa Cruz; #sc-32233; dil. 1:2000) or α-tubulin (Sigma-Aldrich; #T5168; dil. 1:4000).

### 2.11. Statistical Analysis

The distribution of each measurement of in vitro assays was examined for the assumption of normality with the Shapiro–Wilk test. One-way Analysis of Variance (ANOVA), and Bonferroni and Dunnett post hoc analysis are applied to reveal differences between all of the samples, controls, and treated in each cell line, using GraphPad Prism 5 software (GraphPad, San Diego, CA, USA). The alpha level was set at *p* < 0.05.

### 2.12. Clinical Application

Two horses with incompletely resected corneal squamous cell carcinoma were treated under general anesthesia with intracorneal MMC as per the current veterinary literature [17]. At the time of surgery, a session of transpalpebral electroporation was offered to the horses’ owners, as per the current literature [4,18,19,20,21]. Written informed consent was obtained by the horse owners before the administration of the treatment. The horses were sedated with a combination of acepromazine (Prequillan, Fatro, Ozzano Emilia, Italy), butorphanol (Nargesic, ACME, Cavriago, Italy), and romifidine (Sedivet, Boehringer Ingelheim, Milan, Italy) at the doses of 0.03 mg/kg, 0.04 mg/kg, and 0.072 mg/kg, respectively. Premedication was followed by diazepam (Valium, Roche, Milan, Italy) at the dose of 0.05 mg/kg and ketamine (Ketavet 100, MSD, Aprilia, Italy) at the dose of 2.2 mg/kg. At this point, the patients were intubated and kept under general anesthesia with isoflurane (Forane, Abbott, Latina, Italy), as elsewhere described [21].

Briefly, after the surgical excision of the neoplasm, MMC 0.2% solution was injected in the tumor bed, then the eyelids were closed and trains eight biphasic pulses lasting 50 + 50 μsec with 300 μsec interpulse, (total treatment time 3.2 ms) at a voltage of 1000–1300 V/cm, were administered with a clinical electroporator certified for veterinary use (Onkodisruptor^®^). A second session of electrochemotherapy was administered two weeks later as above described.

### 2.13. Histological Analysis

The excised tumor specimens were fixed in 10% buffered formalin and paraffin-embedded. Sections of 5 μm were stained with haematoxylin-eosin, haematoxylin-van Gieson, and PAS-haematoxylin.

## 3. Results

### 3.1. P-gp Expression Profiles of FaDu and CAL 27 Cells

**Figure 1:**
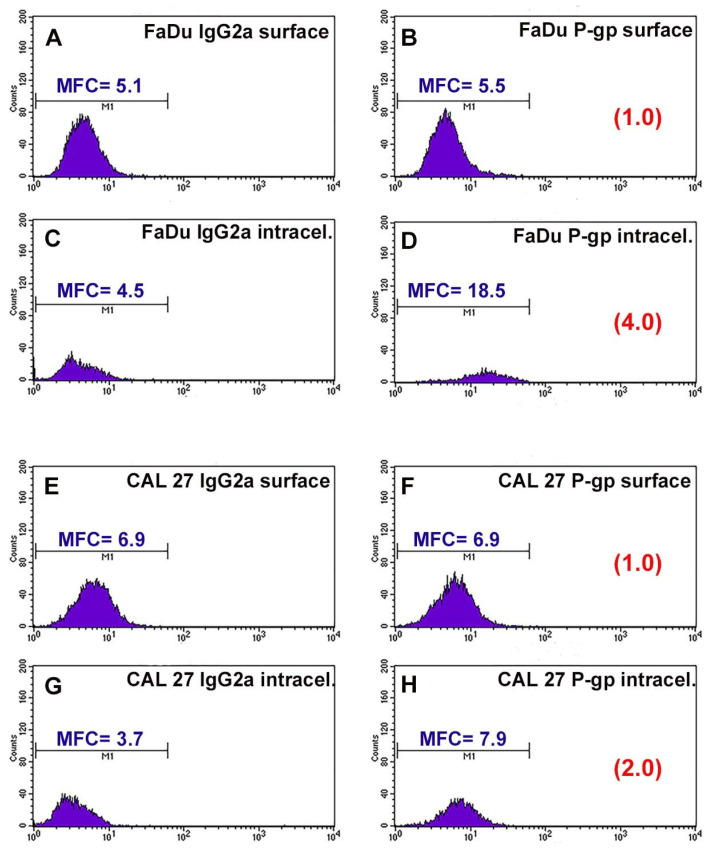
Flow cytometric profiles of surface and intracellular P-gp expression in FaDu and CAL 27 cells. (**A**) FaDu cells labeled with IgG2a globulin on cell surface; (**B**) FaDu cells labeled with MRK16 MAb on cell surface; (**C**) FaDu cells intralabeled with IgG2a globulin; (**D**) FaDu cells intralabeled with MRK16 MAb. (**E**) CAL 27 cells labeled with IgG2a globulin on cell surface; (**F**) CAL 27 cells labeled with MRK16 MAb on cell surface; (**G**) CAL 27 cells intralabeled with IgG2a globulin; (**H**) CAL 27 cells intralabeled with MRK16 MAb. The intensity of the surface P-gp signal was negative on both cell lines, whereas at the intracellular level, FaDu cells (**D**) expressed a higher intensity value than CAL 27 ones (**H**).

P-gp/MDR1 is a common biomarker for MDR, responsible for the drug resistance of several cancer cells. Before studying the chemosensitizing effect of combination therapy, the expression of the surface and intracellular transporter P-gp was analyzed by flow cytometry. Figure 1 shows the flow cytometric profiles obtained after P-gp labeling. The profiles of the FaDu and CAL 27 cells labeled with the MRK16 antibody overlap the respective profiles of the cells labeled with the isotypic control (Figure 1A,B,E,F), demonstrating that both cell lines were completely negative for the surface P-gp.

Instead, it was interesting that the fluorescence profiles obtained for the two cell lines showed a significant difference in the intracellular content of P-gp. FaDu cells were positive for MRK16 MAb, with an approximately 4-fold increase over the isotype control signal (Figure 1C,D). CAL 27 cells also expressed intracytoplasmic P-gp, but to a lesser extent than FaDu cells, with an approximately 2-fold increase over the isotypic control (Figure 1G,H). These results, highlighting the presence of the transport protein at the intracellular level, showed that the two lines we use are intrinsically resistant to common chemotherapeutic agents.

### 3.2. Cells Sensitivity after MMC Treatment Alone for Dose Selection 

**Figure 2:**
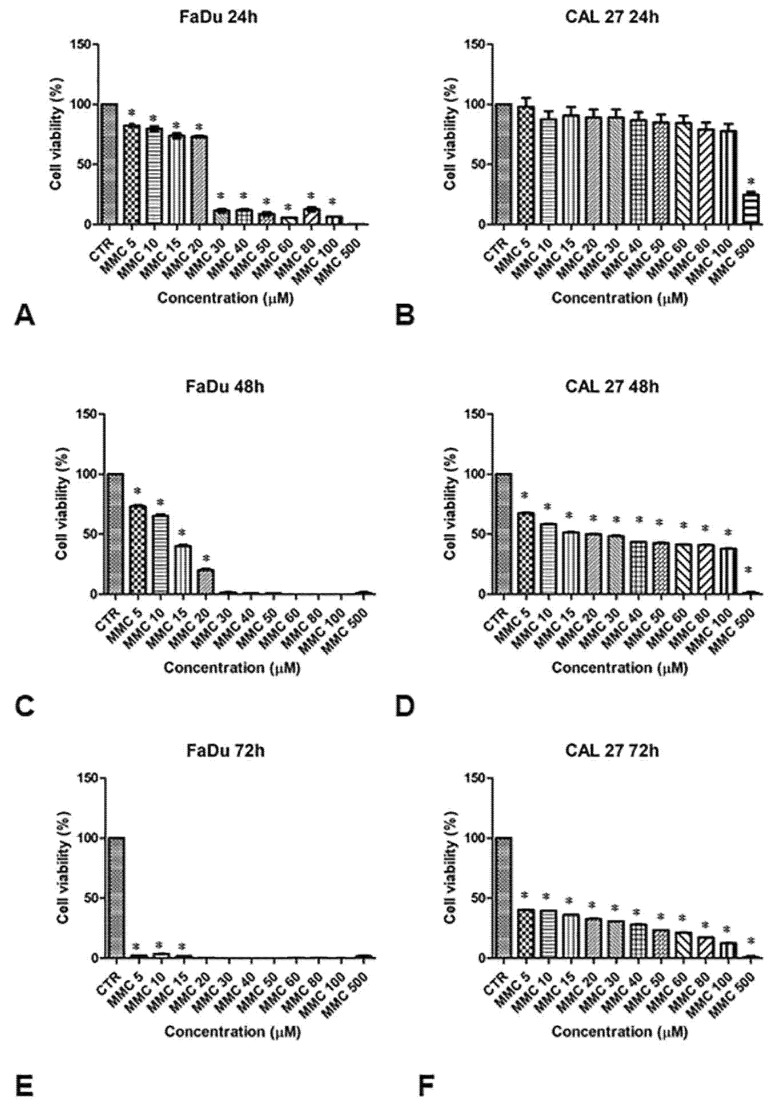
Analysis of the cell viability of FaDu and CAL 27 cells after treatment with mitomycin C (MMC, 5–500 µM). MMT assay was performed on FaDu cells after 24 h (**A**), 48 h (**C**), and 72 h (**E**), and on CAL 27 cells after 24 h (**B**), 48 h (**D**), and 72 h (**F**). The results obtained from three independent experiments, each in sixfold, were expressed as mean ± standard deviation, *: *p* < 0.05 vs. control.

The sensitivity of FaDu and CAL 27 cells to MMC (5–500 µM, for 24, 48, and 72 h) was evaluated by MTT assay, in order to establish the right drug concentration to highlight the chemosensitizing effect of EP. Cell viability in both lines decreased with increasing drug concentrations and exposure time, as shown in Figure 2. The viability of FaDu cells decreases by 20% at an MMC concentration of 10 μM for 24 h. When FaDu cells were treated with MMC concentrations between 30 and 100 μM, cell viability was approximately 15% and was 0% at 500 μM (Figure 2A). Cell viability decreased rapidly in a dose-dependent manner up to 20% with MMC at 20 μM for 48 h (Figure 2C). At 72 h, cell viability was very low (approximately 5%, 5 to 15 μM) (Figure 2E).

CAL 27 cells treated with the same MMC concentrations for 24 h, showed no significant inhibition of cell growth compared to the control cells (Figure 2B). Only at 500 μM was cell viability approximately 30%. At 48 h of treatment, cell viability was between 65% and 40% at 100 μM (Figure 2D). Viability was between 40% and 15% at 72 h (Figure 2F).

These results demonstrate a greater degree of resistance to MMC in the CAL 27 (Figure 2, right panel) compared to FaDu cell line (Figure 2, left panel). Both cell lines were treated with a sub-cytotoxic concentration of MMC (5 µM) for 24 and 48 h to evaluate the chemosensitizing effect of EP.

**Figure 3:**
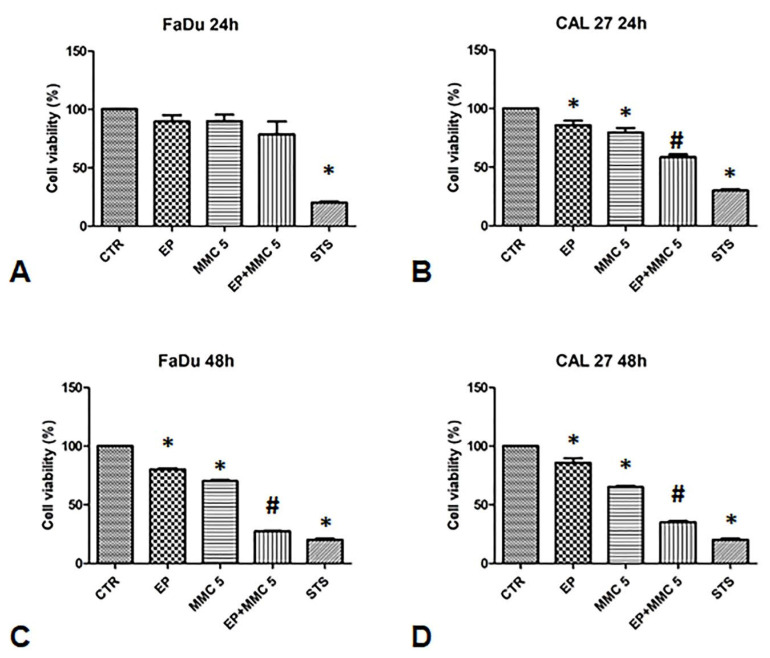
Analysis of the chemosensitizing effect of electroporation (EP) plus MMC on FaDu and CAL 27 cells. MTT assay was performed on FaDu and CAL 27 cells after 24 h (**A**,**B**) and 48 h (**C**,**D**) of: EP alone, treatment with MMC 5 µM alone, combination of EP plus MMC 5 µM, or treatment with staurosporine (STS) 1 µM. The results obtained from three independent experiments, each in sixfold, were expressed as mean ± standard deviation: *: *p* < 0.05 vs. control; #, *p* < 0.05 vs. MMC.

To validate the synergistic effect of combination therapy, cell viability was assessed 24 and 48 h after EP or MMC alone, or combined treatment (EP+MMC) with an MTT test (Figure 3).

When FaDu cells were treated with EP alone for 24 and 48 h, cell viability was 80%; when they were treated with MMC (5µM) it was 80% after 24 h and slightly lower after 48 h (75%). The combined treatment of EP plus MMC (5 µM) showed relative viability of 70% after 24 h, and 35% after 48 h (Figure 3A,C).

In CAL 27 cells, after treatment with EP alone, cell viability was 100% and 90% after 24 h and 48 h, respectively. After treatment with MMC (5 µM) alone, viability was 80% after 24 h and slightly lower after 48 h (70%). When the cells were treated with the drugs combination, viability decreased to 60% after 24 h and 35% after 48 h, as shown in Figure 3B,D.

As a positive control, we used staurosporine which reduced the viability of the FaDu and CAL 27 cells to approximately 30%. These results showed the effectiveness of the combined treatment in both cell lines. Indeed, a 30% viability in FaDu cells and 40% in CAL 27 was observed compared to MMC alone treatment, suggesting that electrical pulses changed the permeability of the cell membrane by increasing the cytotoxic effect of MMC.

### 3.3. Observation of the Cytotoxic Effect of the EP+MMC Combination by Optical Microscopy

The effects, evaluated by the MTT assay, were confirmed by phase contrast microscopic observations.

**Figure 4:**
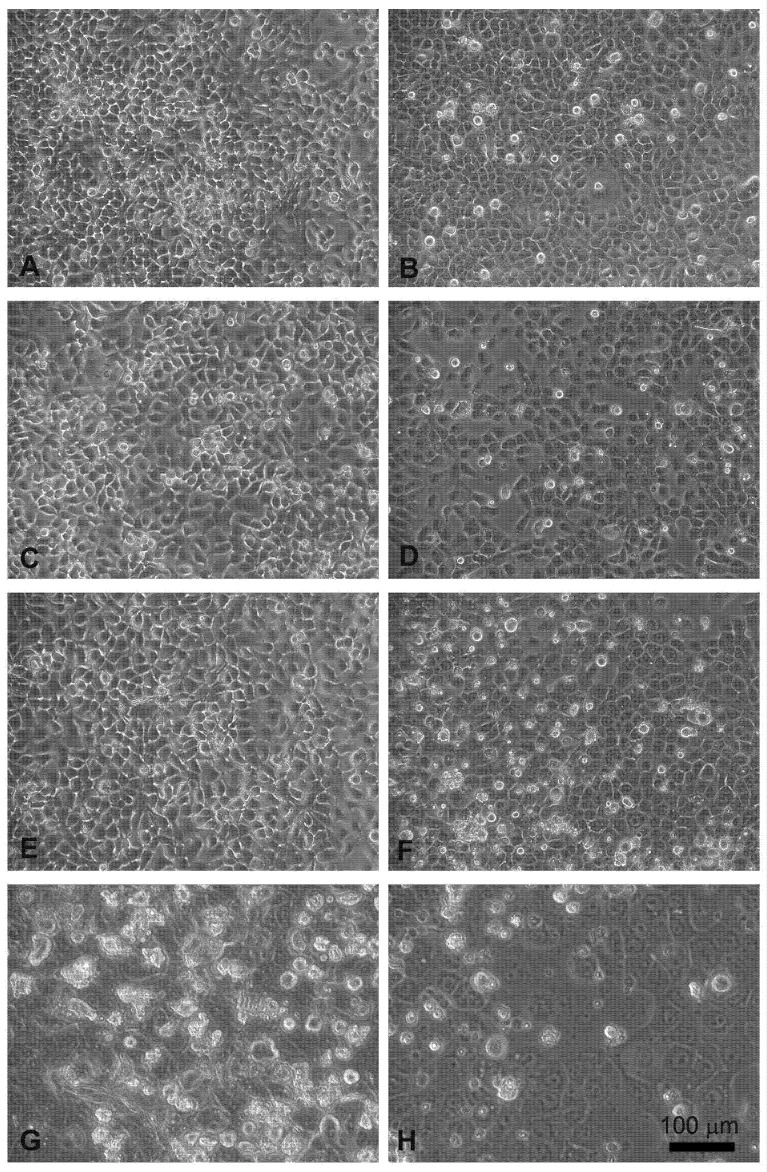
Morphological changes in FaDu cells (left panel) and CAL 27 cells (right panel) induced by EP and MMC after 24 h of treatment. (**A**,**B**) Control, no treatment; (**C**,**D**) single EP; (**E**,**F**) 5µM MMC; (**G**,**H**) combined treatment (EP + MMC).

Head and neck squamous carcinoma cell lines grow adherent to the substrate, appear flat, elliptical (FaDu cells, Figure 4A), or polygonal, and highly granular (CAL 27 cells, Figure 4B). After 24 hours of treatment with electroporation (Figure 4C) or MMC (5 µM) (Figure 4E) the FaDu cells were similar to the control. In the combined treatment (Figure 4G) many cells were detached from the substrate and the adherent ones were morphologically modified compared to the control (Figure 4A).

Similar results were obtained on CAL 27 cells, the electroporated or MMC-treated cells showed no evident alterations (Figure 4D,F), while in the combined EP + MMC treatment a severely modified cell morphology appeared (Figure 4H). There were more detached cells; those adhering to the substrate had larger nuclei and cells. These observations supported the MTT data confirming the cytotoxic enhancing effect of electroporation when combined with MMC on drug-resistant cell lines.

### 3.4. Morphological Changes Revealed by SEM Observations

**Figure 5:**
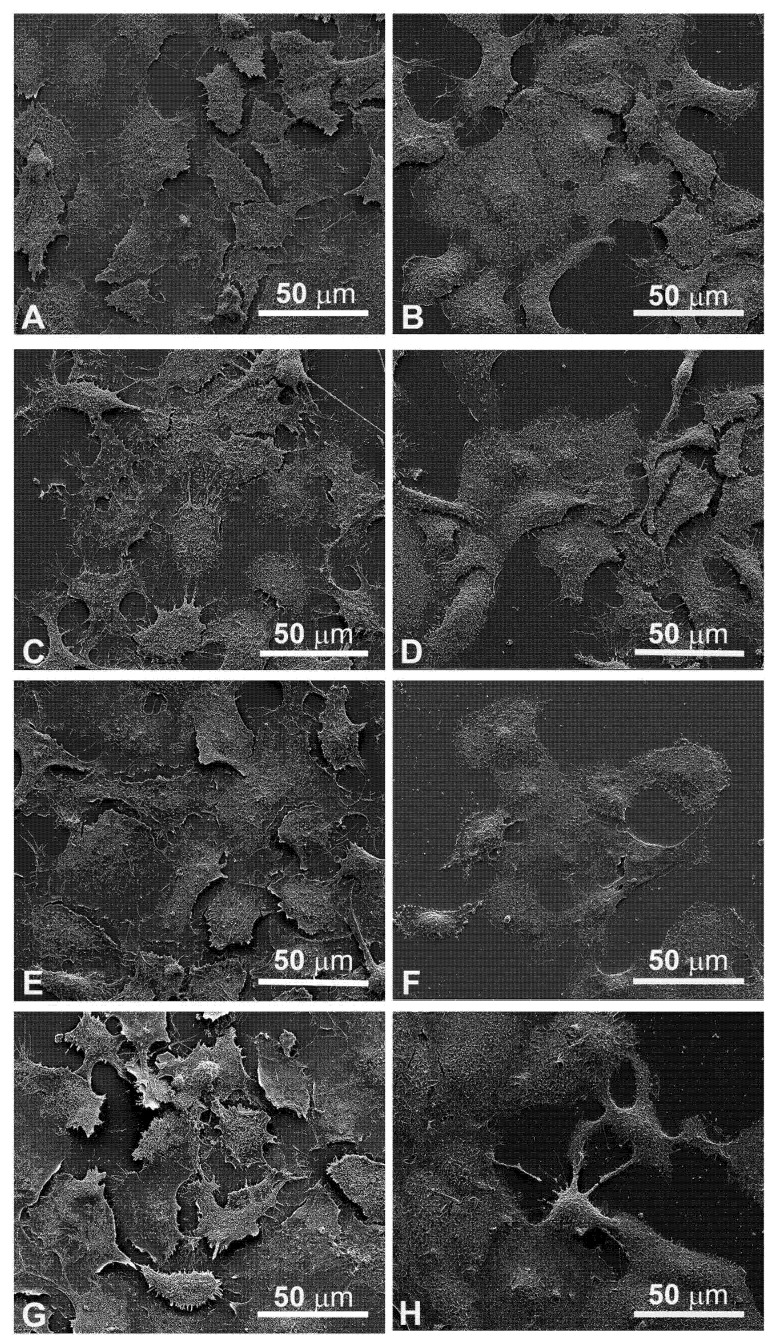
Scanning electron microscopy observations of FaDu cells (left panel) and CAL 27 cells (right panel) for 24 h after treatment. (**A**,**B**) Control, no treatment; (**C**,**D**) single EP; (**E**,**F**) MMC (5 µM); (**G**,**H**) combined treatment (EP + MMC).

**Figure 6:**
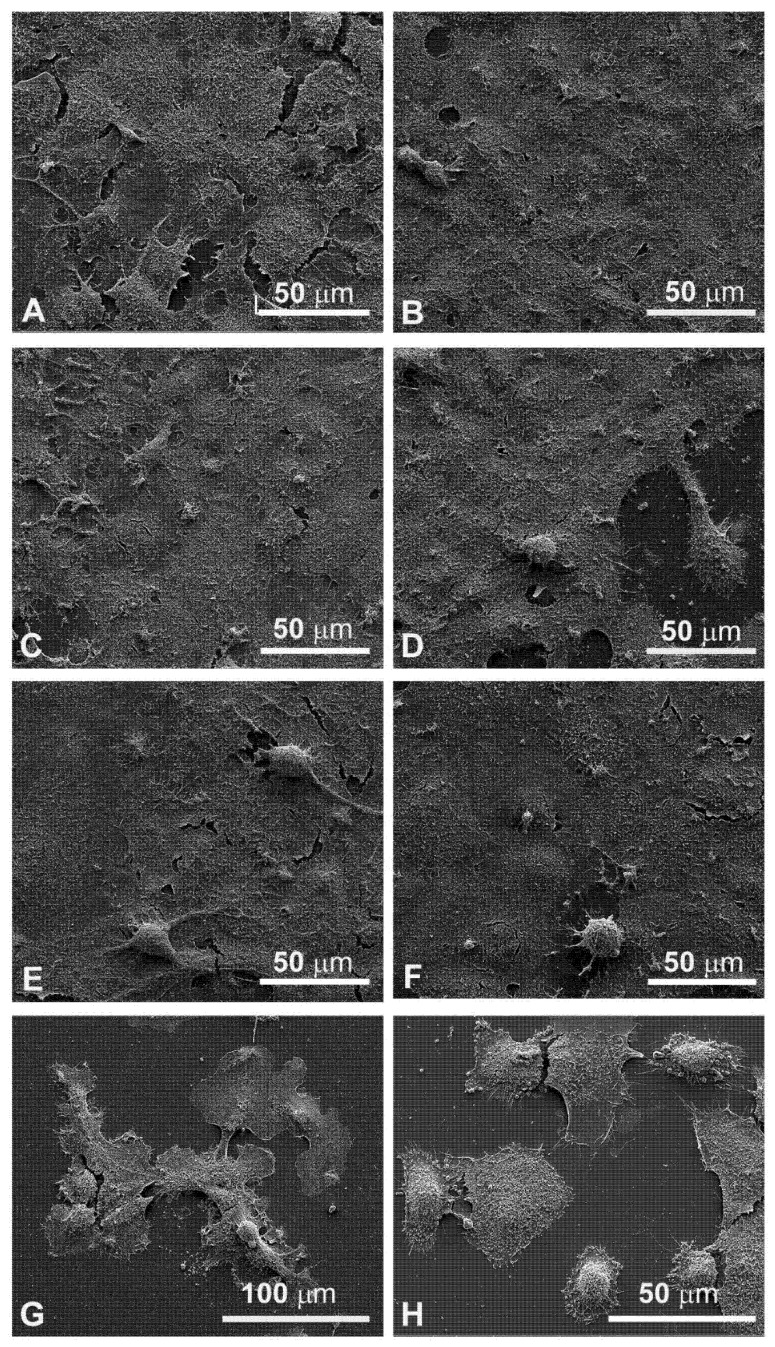
Scanning electron microscopy observations of FaDu cells (left panel) and CAL 27 cells (right cells) for 48 h after treatment. (**A**,**B**) Control, no treatment; (**C**,**D**) single EP; (**E**,**F**) MMC (5 µM); (**G**,**H**) combined treatment (EP + MMC).

Since the cellular shape is related to its function, careful observations of morphological changes were made after electroporation and MMC treatments by SEM. The control FaDu cells had variable shapes (Figure 5A), while the CAL 27 cells were mainly polygonal (Figure 5B), both had microvilli randomly distributed on the surface. Single treatment of FaDu cells with EP for 24 and 48 hours (Figure 5C and Figure 6C) did not induce cytotoxicity compared to the control cells (Figure 5A and Figure 6A). After MMC treatment for 24 h, FaDu cells were normal in shape, size, and microvilli, no irregular extensions were detected, and the cell membrane was intact (Figure 5E). After 48 h, signs of cellular stress appeared (cell rounding and initial detachment from the substrate, Figure 6E). The electroporated cells treated with MMC for 24 h (Figure 5G) or 48 h (Figure 6G) showed evident morphological alterations in relation to the prolongation of the treatment time (rounding, detachment from the substrate, inhomogeneity of the microvilli).

Morphological modifications were also observed in CAL 27 cells. A single treatment with EP for 24 h and 48 h (Figure 5D and Figure 6D) and MMC for 24 h (Figure 5F) or 48 h (Figure 6F) was similar to the control (Figure 5B and Figure 6B). With combined treatment (EP and MMC for 24 h), cells showed fewer signs of cellular damage (Figure 5H) compared to 48 h (Figure 6H). CAL 27 cells were partially detached and many of them were highly modified by treatments compared to the single exposures (EP or MMC).

**Figure 7:**
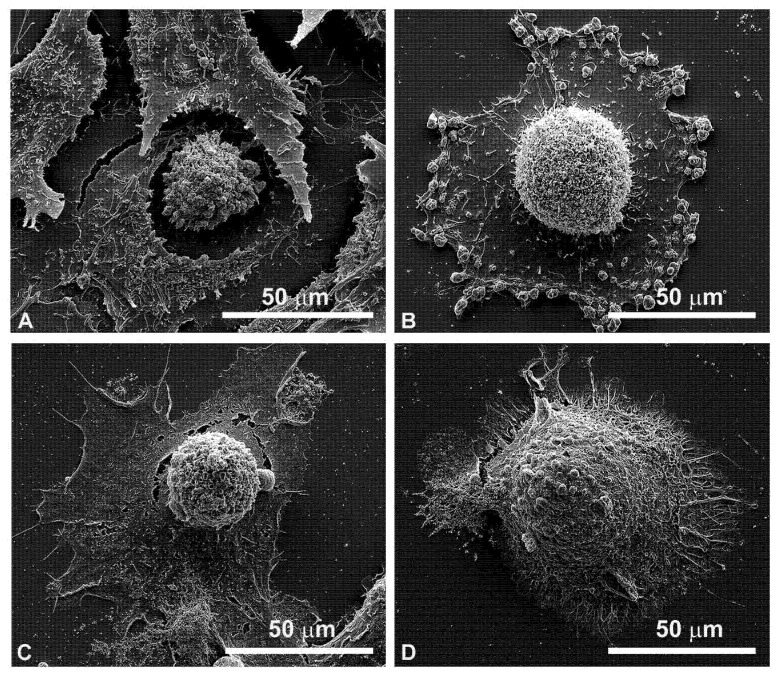
Details of individual cells by scanning electron microscopy after combined treatments (EP + MMC). (**A**) FaDu cells after 24 h; (**B**) CAL 27 cells after 24 h; (**C**) FaDu cells after 48 h; (**D**) CAL 27 cells after 48 h.

Figure 7 shows the enlarged details of highly modified cells by EP plus MMC treatment in FaDu cells (Figure 7A for 24 h, Figure 7C for 48 h) and in CAL 27 (Figure 7B for 24 h and Figure 7D for 48 h). Notable signs of cell retraction are observed leading to cell ruptures, nuclear-localized swelling, and numerous bubbles of different sizes on the cell surface, suggesting the involvement of the apoptotic process.

### 3.5. Apoptosis Detected by Flow Cytometry

**Figure 8:**
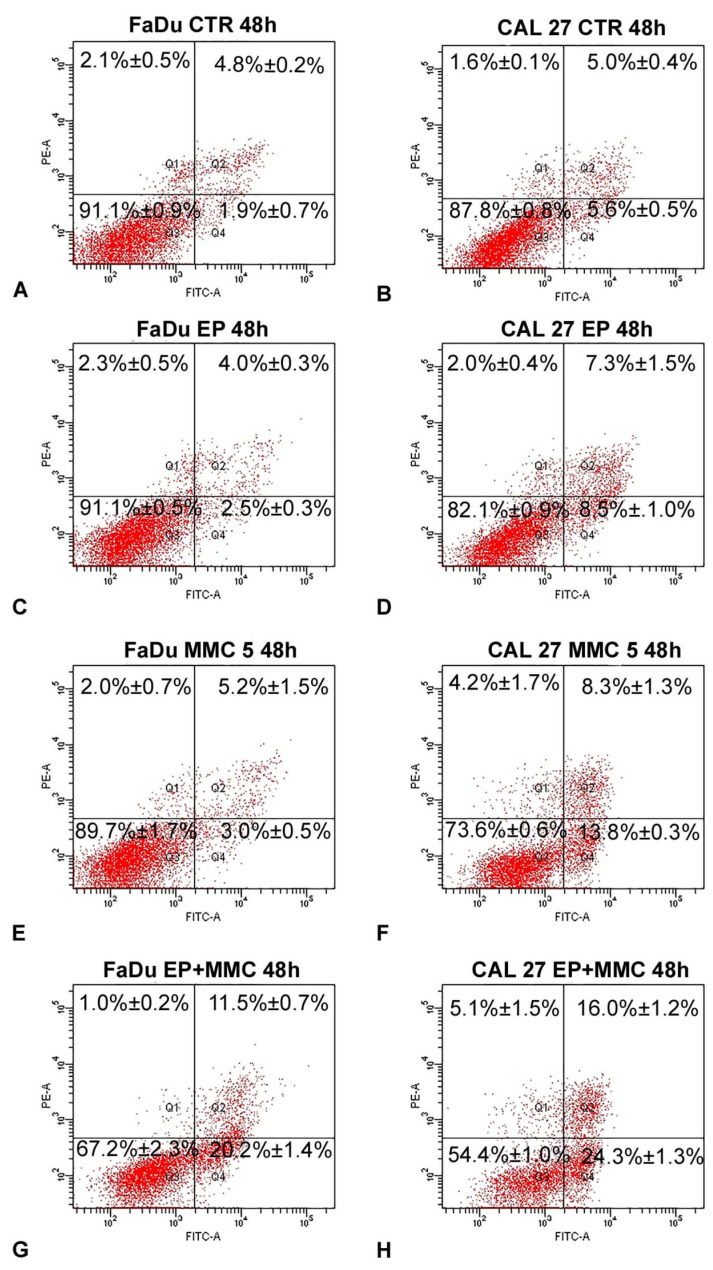
Representative dot plots of apoptosis induction on FaDu and CAL 27 cells after Annexin V/PI dual staining. (**A**,**B**) Control cells; (**C**,**D**) treated with EP; (**E**,**F**) MMC (5 µM for 48 h); (**G**,**H**) combined treatment (EP+MMC). Dot plots are representative of three independent experiments; values indicate mean ± standard deviation.

To determine if combined therapy (ECT) triggered cell death by apoptosis, surface exposure of phosphatidylserine, an early event of the apoptotic program, was assessed using the Annexin V/FITC-PI assay by flow cytometry. The results of this analysis, carried out after the treatment of FaDu and CAL 27 cells with MMC (5 µM), alone or in combination with biphasic pulse trains (eight 50 + 50 μs pulses), for 48 h are shown in Figure 8. As demonstrated by a previous assay, EP did not change the apoptotic and necrotic fractions of either cell line (Figure 8C,D) in comparison to the control samples (Figure 8A,B). The single treatment of FaDu cells with MMC (5 µM) did not significantly change the early apoptotic fraction (3.0%±0.5%, Figure 8E) and the late apoptotic fraction (5.2% ± 1.5%, Figure 8E), which were comparable with the control cells (1.9 ± 0.7% and 4.8% ± 0.2%, Figure 8A). A slight increase of the early apoptotic fraction (13.8% ± 0.3%, Figure 8F) was revealed after CAL 27 treatment with MMC (5 µM) versus the 5.6% ± 05% fraction of the control cells (Figure 8B). Instead, the association of EP plus MMC caused an increase of the cell percentages of apoptotic fractions: FaDu cells showed an increase in the early apoptotic fraction (20.2% ± 1.4% versus 3%.0 ± 0.5% of MMC-treated cells) and an increase in the late apoptotic cells (11.5% ± 0.7% versus 5.2% ± 1.5% of MMC treated cells). The same effects were obtained in CAL 27 cells: an increase of the early apoptotic fraction (24.3% ± 1.3% versus 13.8% ± 0.3% of MMC-treated cells) and of the late apoptotic fraction (16.0% ± 1.2% vs. 8.3% ± 1.3% of MMC-treated cells) was observed (Figure 8H). In both cell lines, the necrotic fraction was significantly lower than the apoptotic one.

These results demonstrated that EP sensitized head and neck squamous cell carcinoma (HNSCC) cells to MMC action by apoptosis induction.

### 3.6. Immunofluorescence Imaging of Cytochrome C Release for Apoptotic Studies

**Figure 9:**
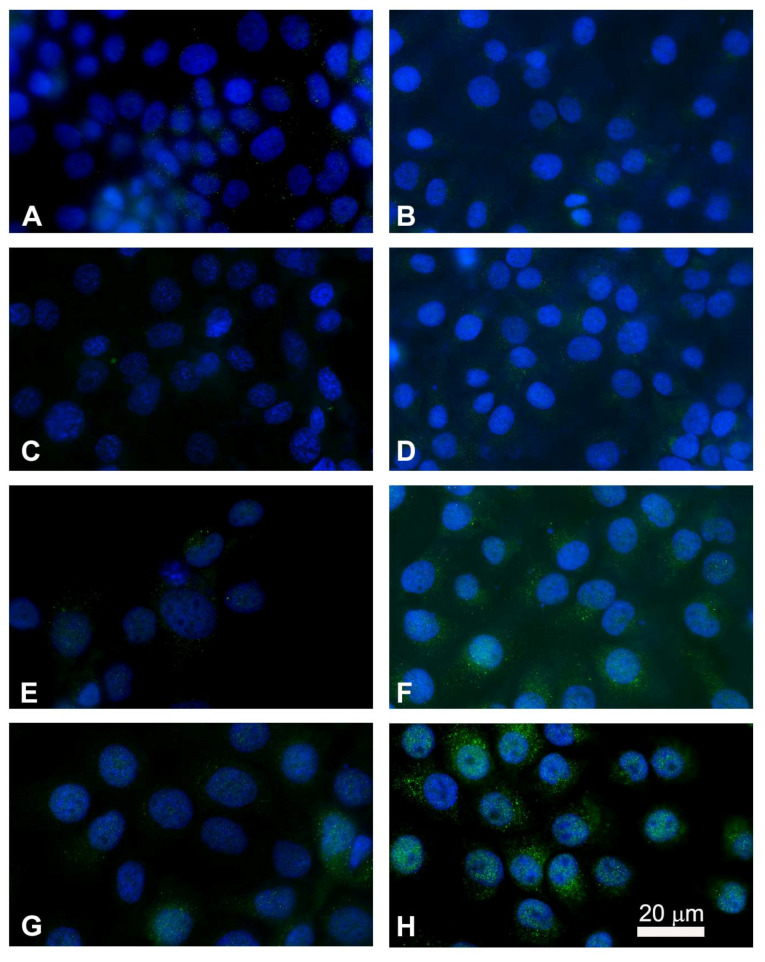
Immunostaining of cytochrome c with nuclear Hoechst staining in FaDu cells (left panel) and in CAL 27 cells (right panel). Intracellular distribution of cytochrome c (green) and nuclei localization (blue) were performed by fluorescence microscopy. (**A**,**B**) Untreated cells; (**C**,**D**) single electroporated cells; (**E**,**F**) cells treated with MMC alone (5 µM for 24 h); (**G**,**H**) cells treated with the combined therapy (EP + MMC). Point structures in green, localized in the cytoplasm, highlight the cytochrome c released.

After observing the ultrastructural changes in both FaDu and CAL 27 cell lines and quantifying the rate of apoptosis after EP plus MMC treatment, we examined the localization by marking cytochrome c in green and the nucleus blue (Hoechst staining) by immunofluorescence. Cytochrome c is released from the inner membrane of the mitochondria into the cytoplasm as a result of an apoptotic stimulus. In untreated cells, cytochrome c showed punctate cytoplasmic staining, which is in agreement with its localization in the mitochondria (Figure 9A,B). Electroporation did not alter the fluorescent signal compared to the control cells (Figure 9C,D). Treatment with MMC showed an increase in bright spots in the cytoplasm indicating the partial translocation of cytochrome c to the cytoplasmic level (Figure 9E,F). The combined treatment induced a significant release of cytochrome c in both FaDu and CAL 27 cells (Figure 9G,H) compared to treatment with MMC alone (Figure 9E,F). The greater effect observed in CAL 27 cells (Figure 9H) confirms the quantitative analysis of apoptosis.

### 3.7. Analysis of Apoptotic and Autophagic-Associated Proteins Expression

**Figure 10:**
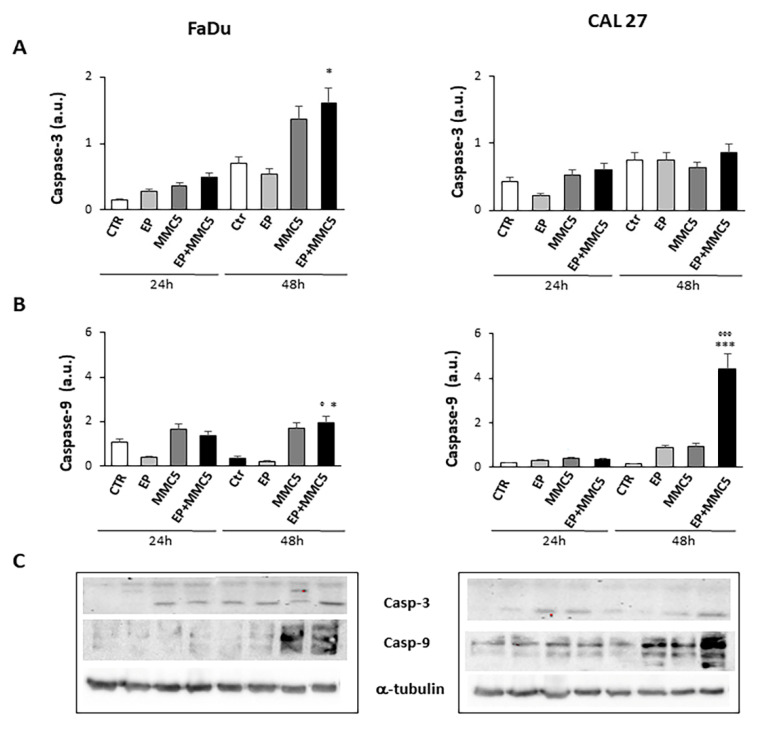
Caspase expression. The bar graphs show the relative quantification of caspase-3 (**A**) and caspase-9 (**B**) densitometry in the FaDu (left panel) and in the CAL 27 cells (right panel). Each protein is normalized to α-tubulin. Data are expressed as the mean ± SD of three independent experiments. * *p* ≤ 0.05 vs. control; ° *p* ≤ 0.05 compared to MMC. *** *p* < 0.001 vs. control; °°° *p* ≤ 0.001 compared to MMC. (**C**) Representative Western blot analysis of caspase-3 and -9 in FaDu (left panel) and CAL 27 cells (right panel). The α-tubulin determination was used as load control. Not-cropped Western blot figures are available in Figure S1.

In order to assess the effect of combined therapy (EP + MMC), caspase-3 and -9, involved in the apoptotic process, were examined by Western blot analysis (Figure 10A,B). The combined treatment induced more expression of caspase-3 in both FaDu and CAL 27 cells compared to treatment with MMC alone (Figure 10A). In particular, in CAL 27 cells 48h after treatment there was a visibly greater effect, which was confirmed by analysis with Annexin V and by cytochrome c release.

**Figure 11:**
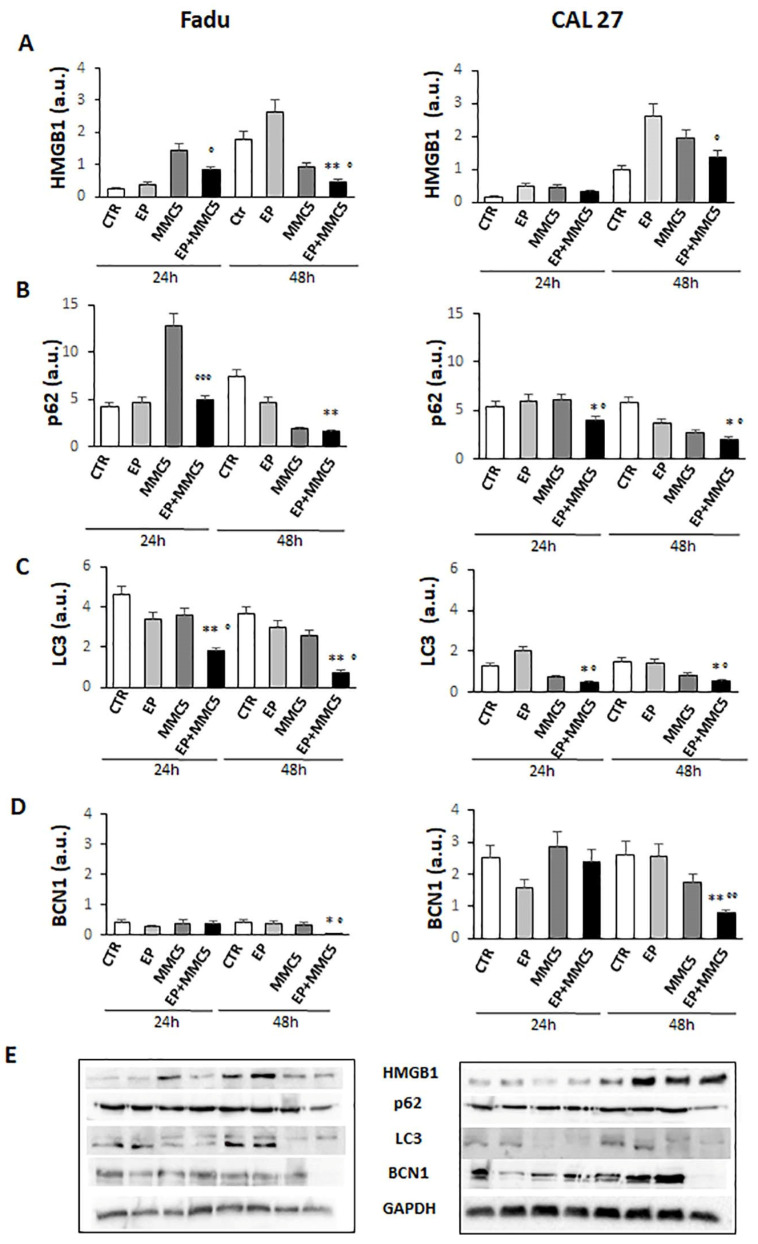
Autophagic pathway. The bar graphs show the quantification of relative densitometry of HMGB1 (**A**), p62 (**B**), LC3 (**C**), and BCN1 (**D**) in the FaDu (left panel) and CAL 27 cells (right panel). Each protein is normalized to GAPDH. Data are expressed as the mean ± SD of three independent experiments. * *p* ≤ 0.05 vs. control; ° *p* < 0.05 with respect to MMC; ** *p* ≤ 0.01 vs. control; °° *p* ≤ 0.01 compared to MMC; °°° *p* ≤ 0.001 compared to MMC (**E**). Representative Western blot analysis of all proteins in FaDu (left panel) and CAL27 cells (right panel). The GAPDH determination was used as load controls. Not-ropped Western blot figures are available in Figures S2 and S3.

The effects of the combined treatment are also in line with the decreased levels of the high-mobility group box 1 (HMGB1) protein (FaDu and CAL 27 cells at 24 h and 48 h, Figure 11A), known to be a nuclear protein that regulates gene expression and nucleosome stability. This protein has an abnormal expression in different tumors including glioma, pancreatic cancer, and epithelial ovarian cancer, and promotes autophagy by exerting significant functions for survival [22].

**Figure 12:**
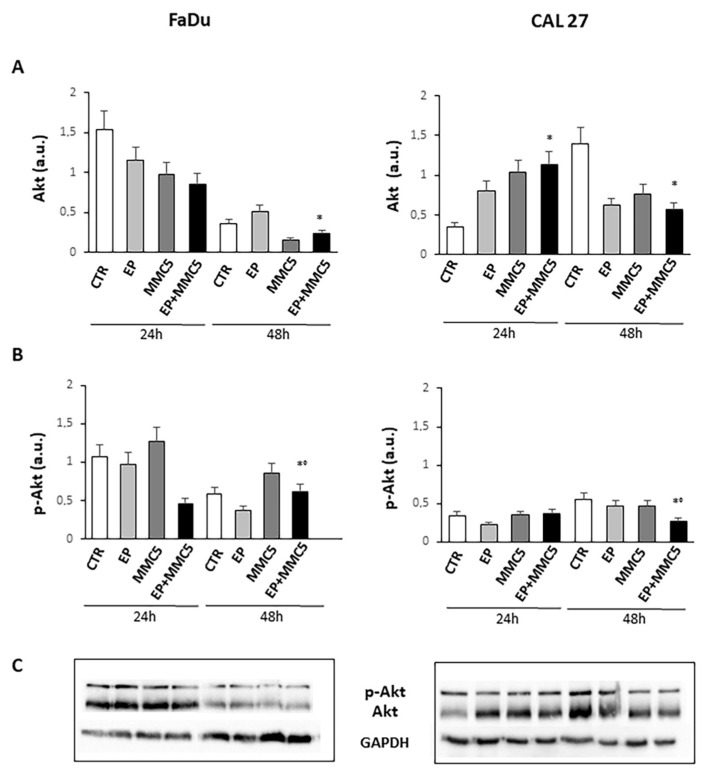
Evaluation of Akt and phospho-Akt. The bar graphs show the quantification of the relative densitometry of Akt (**A**) and phospho-Akt (**B**) in the FaDu (left panel) and CAL 27 cells (right panel). Each protein is normalized to GAPDH. Data are expressed as the mean ± SD of three independent experiments. * *p* ≤ 0.05 vs. control; ° *p* ≤ 0.05 compared to MMC. (**C**) Representative Western blot analysis of Akt and phospho-Akt in the FaDu (left panel) and CAL 27 cells (right panel). The GAPDH determination was used as load control. Not-cropped Western blot figures are available in Figure S4.

Because the phosphorylation and activity of Akt are implicated not only in cell growth but also in the mechanism of drug resistance and autophagy, we analyzed Akt phosphorylation at S473 (Figure 12). We found that prolonged combined treatment (EP+MMC) significantly reduced the phosphorylation of protein in either cell line compared to treatment with MMC alone. Moreover, in either cell line the expression of Akt decreased after prolonged combined treatment (Figure 12).

Given Akt’s involvement in autophagy, we examined the expression levels of some proteins involved in the different steps of the autophagic process, p62, LC3s, and Beclin1 (BNC1). As shown in Figure 11B–D, the levels of the p62, LC3s, and BCN1 notably decreased in both cell lines after combined treatment (EP + MMC), regardless of the duration of treatment.

### 3.8. Clinical Results

**Figure 13:**
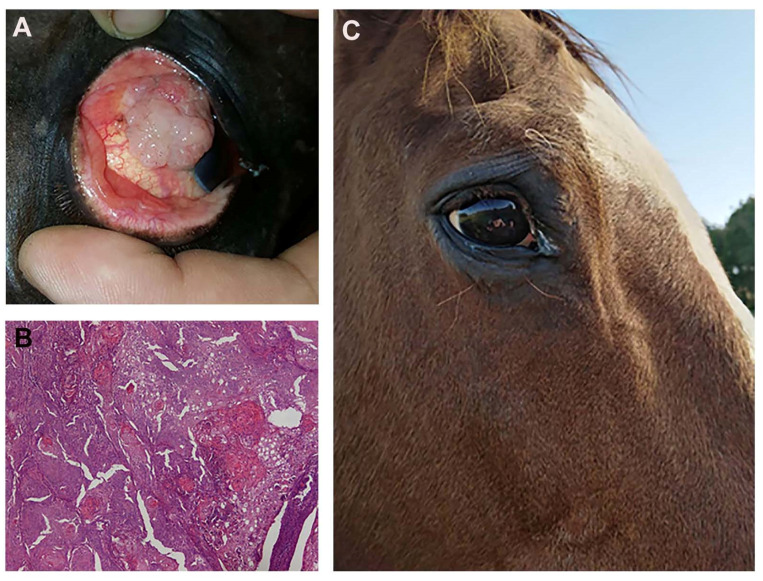
(**A**) a fourteen-year-old saddlehorse with corneal squamous cell carcinoma, resulting in a proliferation that covers most of the corneal surface. (**B**) Histopathology after surgical excision: a high tumor cell mitotic index and the formation of several keratin pearls are visible (Hematoxylin and Eosin staining; original magnification ×20). (**C**) The patient at one year follow up: there is no evidence of recurrence and eye functionality has been fully restored.

Both horses tolerated the two ECT sessions without side effects to the eye globe, the tumor is still in complete remission after 14 and 18 months, respectively. Figure 13 shows the outcome of one of the equine patients: A) Patient at presentation, B) the histopathological appearance of the corneal squamous cell carcinoma, and C) the patient at one year follow up.

## 4. Discussion

Multidrug resistance (MDR) derives from the multiple defense mechanisms that cancer cells adopt towards clinical treatments. Unfortunately, many treatments also fail due to the severe side effects induced by chemotherapy, such as fatigue, loss of appetite, hair loss, infections, sore mouth, bleeding, and anemia which results in a temporary interruption of therapy. Combination therapy has often proved more effective than single-modal treatment. The goal for overcoming drug resistance is to provide targeted therapies for each type of tumor and reduce cellular effects that are damaging to healthy tissue. The use of electrochemotherapy (ECT) gives the possibility of intervening with adjuvant therapy in combination with chemotherapy. During electroporation (EP), external pulsed electric fields are applied to cells or tissues, increasing the electric potential across the cell membrane. Increasing the potential facilitates the creation of aqueous nanoscale pores in the lipid bilayer, increasing the permeability of the cell membrane to macromolecules. If the effect is transient and the pores are resealed, it is called a reversible EP [23]. Several factors contribute to the difference in effect, including cell size, tissue type, and electrical pulse parameters: number of pulses, amplitude, frequency, duration, and shape [24]. With the application of electrical pulses, in addition to the transient permeabilization of the plasma membrane, modifications of the endomembranous system are also observed [25]. The alteration in the organization of the intracellular membrane organelles avoids the entrapment of the drug in them and therefore favors a greater availability of the target inducing a reduction of MDR [26]. Most adverse effects of MMC are dose-related, including myelosuppression, nausea, vomiting, diarrhea, stomatitis, dementia, and alopecia. Hence, it is very important to reduce the dose of MMC as it causes some negative effects which are unavoidable with this therapy.

To evaluate and investigate the main mechanisms related to the combined electroporation and MMC treatments in the cell cultures used in this study, a careful analysis of P-gp expression on cell membranes was performed. Both cell lines (FaDu and CAL 27) did not express surface P-gp, while a difference in the intracellular protein content was observed between the two lines. As previously shown, P-gp in intracellular vesicles plays a fundamental role in the regulation of intracellular drug trafficking, sequestering it away from the target and transporting it outwards [27]. The FaDu line had twice the percentage of intracellular P-gp expression compared to the CAL 27 line, both of which are inherently drug-resistant [28].

A first analysis, before doing the experiments in combination with electroporation, was to evaluate the right dose of MMC for the two cell lines.

The strategy was to use a sub-cytotoxic dose of MMC to demonstrate the potentiating effect of electroporation on MMC cytotoxicity in combination treatments.

Figure 2 shows a different sensitivity in dose-response for the same treatment time in the two cell lines, CAL 27 cells were more resistant to MMC than FaDu, despite the presence of lower intracytoplasmic P-gp expression (Figure 1). The lower toxicity observed in MMC treatments on CAL 27 cells, could be due to the different resistance mechanisms demonstrated in this cell line independent of the intracellular expression of P-gp. CAL 27cells overexpress the αvβ3 integrin which confers resistance to several drugs including MMC through the loss of pSrc (Y418) [29]. The MMC concentration chosen for the two lines was 5µM for 24 and 48 h. Quantitative analysis of the cytotoxic effect of the single (EP or MMC) and the combined (EP + MMC) treatments revealed significant inhibition of cell growth in FaDu at 48 h with EP + MMC treatment equal to 70% and a reduction of 60% of the CAL 27 cell line (Figure 3). The single treatments reduced tumor cell viability much less than the combinations in both lines.

The quantitative cytotoxic analysis was further confirmed by observation both in light microscopy (Figure 4) and in scanning electron microscopy (Figure 5). The morphology in both situations agreed with the experimental data previously obtained; in both cell lines, the morphology was significantly altered when treated in combination EP+MMC, compared to the single treatments. These results confirmed a close link between efficacy in response to treatments and ultrastructural morphological behavior of the cells. 

From the details in the scanning electron microscopy (Figure 7), the presence of clear apoptotic figures was observed.

Quantitative analysis of the presence of apoptosis in the two cell lines was then performed. Analysis of phosphatidylserine exposure in the plasma membrane of the FaDu and CAL 27 cells by flow cytometry, showed the induction of programmed cell death, in combined EP+MMC treatments (Figure 8).

The quantitative evaluation revealed that the single treatments (EP, MMC) showed the percentage of cells in apoptosis in a manner comparable to the controls.

In the combined treatments, the rate of apoptosis was highly significant in both cell lines, demonstrating that the enhancement of the EP-induced cytotoxic and antiproliferative effect on MMC observed in Figure 3 was due to the induction of apoptosis.

As can be seen at the beginning of our experimental design to demonstrate the potentiating effect of EP on the MMC efficacy we used the MTT assay (Figure 3) involving the mitochondrion and its activity. The mitochondria play a fundamental role in the initiation of triggering apoptosis.

During this phase, the release of various proteins from the mitochondrial intermembrane space to the cytosol is observed, including cytochrome c.

The observation under the fluorescence microscopy highlighted the release of cytochrome c at the cytoplasmic level, confirming again the onset of programmed cell death in the combined treatments (EP + MMC) in both cell lines (Figure 9). The activated PI3K/Akt pathway is known to induce phosphorylation of various proteins, including P-gp, which induce drug resistance mechanisms by favoring tumor growth, autophagy, inhibition of apoptosis, and promoting invasion and metastasis. MDR is often associated with the anomalous activation of this pathway which cooperates with different targets thus providing survival signals for different chemotherapeutics [30]. By analyzing the Akt phosphorylation in our intrinsically MDR cell lines, we found that combined treatments (EP+MMC) significantly reduced its activity while simultaneously reducing the non-phosphorylated form of Akt. These results were in agreement with the literature data on the efficacy of the use of electroporation with some chemotherapy regimens [31,32].

After showing the onset of apoptosis, the significant decrease in Akt phosphorylation and release of cytochrome c by mitochondria in the combined treatments of EP+MMC, we analyzed the proteolytic caspase cascade, which is the executor of intrinsic apoptosis through the expression of caspase-9 and caspase-3. Caspase-9 is known to act by decoupling mitochondria for increased ROS content, while caspase-3 inhibits the production of ROS which is essential for efficient intrinsic apoptosis [33]. We then analyzed by Western blot the activation of the caspase-9 initiator and caspase-3 executing enzyme when CAL 27 and FaDu cells were treated with single and combined selective stimuli (EP, MMC, EP + MMC). A marked increase in caspase-9 expression was observed in the combined treatment at 48 h in CAL 27 cells and a minor increase in FaDu cells; however, it was greater in 48 h than in 24 h. Caspase-3 is known to play a central role in the execution phase of apoptosis [34]. Having therefore observed a significant increase in their expression compared to single treatments, at 24 h and 48 h in both cell lines, we concluded that the combination of a mechanical pulse, adapted to the experimental conditions, and sub-cytotoxic doses of MMC, can promote apoptosis under intrinsic MDR conditions.

Many cytotoxic drugs, including MMC, activate apoptosis but also autophagy, as a mechanism of resistance [13,35]. For cancer cells, the activation of autophagy may be a stimulus for survival from drug-induced bioenergetic stress. Furthermore, with the activation of autophagy, cells have the possibility of eliminating unfolded proteins and damaged organelles, and thus, obtain the benefits of the generation of glycolytic substrates [36].

The HMGB1 protein can regulate autophagy in response to drug treatment and thus influence its efficacy [22]. It has already been shown that when HMGB1 is in the nucleus, it increases the expression of HSP27 to induce autophagy [37], while in the cytoplasm, it activates the Beclin1/PI3K-III complex which promotes autophagy [38,39].

Furthermore, the inhibition of autophagy by small molecules (RNAi) reduced the release of HMGB1, increasing efficacy and promoting apoptosis [38]. Xu et al. showed that the regulation of non-coding RNAs can reverse chemoresistance by inhibiting HMBG1 which induced autophagy in cancer cells [22]. In our cellular models, we obtained a decrease in HMGB1 protein expression after combined treatment (EP + MMC) at both 24 h and 48 h compared to the single treatments (Figure 11A). These results indicated an inhibitory role of EP in the autophagic survival mechanism induced by MMC treatment. At this point, we explored the fundamental parameters that could determine the presence, and potentially the inhibition, of autophagy by means of EP, such as the expression of the Beclin1, p62, and LC3 proteins.

In the complex mechanism by which autophagy is activated, Beclin1 plays an important and necessary role in the formation of autophagic vesicles and its level can determine whether or not the cell will enter autophagy [40]. Another important factor for the implementation of the autophagic process is the cytoplasmic form of the protein light chain associated with microtubules 3 (LC3-I, 16 kDa) which is activated, transferred, and converted into the phosphatidylethanolamine (PE)–conjugated form, LC3-II (14 kDa), associated with the membrane, and finally recruited into the autophagosomes [41]. The relationship between p62 and the proteins that regulate the mechanisms of apoptosis and autophagy is complex to define. Basal autophagy under normal conditions has relatively low levels of p62 because it is degraded with its target by the lysosomal system. However, there are some resistant tumors such as cisplatin-resistant ovarian epithelial carcinoma that overexpress the baseline level of p62, and reducing its levels increases drug sensitivity [42]. In esophageal squamous cell carcinoma tissues, p62 levels were found to be upregulated through the activation of the protein kinase C (PKC) pathway, the enhanced p62 protein level induced cellular resistance to apoptosis and cell growth in tumors [43]. Additionally, in breast cancers, p62 is related to the advanced clinical stage and high protein expression is associated with the onset of distant metastasis and aggressive features [44]. Based on Beclin 1, LC3II/LC3I, and p62 expression results, being considered the main factors for the assessment of autophagy levels and progression, we can state that EP is capable of inducing apoptosis inhibiting the autophagy mechanism induced by chemotherapy with MMC on human hypopharyngeal carcinoma (FaDu) and carcinoma of the tongue (CAL 27) cell lines. Finally, in the two veterinary patients in which the combination of EP and MMC was used to increase local control after the marginal resection of corneal squamous cell carcinoma, the therapy was well tolerated, with no direct damage to the corneal tissue or visual impairment. Furthermore, the association increased local control in both patients, showing the clinical feasibility of this approach. Further investigations are needed to validate this clinical approach, in larger cohorts of patients.

## 5. Conclusions

Initially, our work focused on identifying the instrumental parameters of EP to promote MMC uptake in the cell membranes of highly aggressive tumors such as pharyngeal and tongue carcinomas. EP, with individually defined parameters, has a reversible, and no harmful, effect on normal cells. We also identified the resistance mechanisms of MMC in these tumors and overcame them by using it in combination with PE.

After an in-depth study of the main cell death mechanisms, we can affirm that the effect by which EP increases the cytotoxic effect of MMC is due to the inhibition of the protective mechanism of autophagy in favor of apoptosis.

In conclusion, we suggest that this study may have significance in experimental applications in vivo since for the first time it provides experimental evidence on the use of the electrical pulse technique to inhibit the autophagic process used by cancer cells for survival.

## Data Availability

The datasets analyzed during the current study are available from the corresponding authors upon reasonable request.

## Supplementary Materials

The following are available online at https://www.mdpi.com/article/10.3390/cancers13153867/s1, Figure S1: Not-cropped Western blot figures for Figure 10; Figure S2 and Figure S3: Not-cropped Western blot figures for Figure 11; Figure S4: Not-cropped Western blot figures for Figure 12.
