# Supplementary figures and images for: Electrochemotherapy with Mitomycin C Potentiates Apoptosis Death by Inhibiting Autophagy in Squamous Carcinoma Cells
Source: Cancers (Basel). 2021 Jul 31;13(15):3867. doi: 10.3390/cancers13153867 (PMC8345561; doi:10.3390/cancers13153867)

**Figure S1**

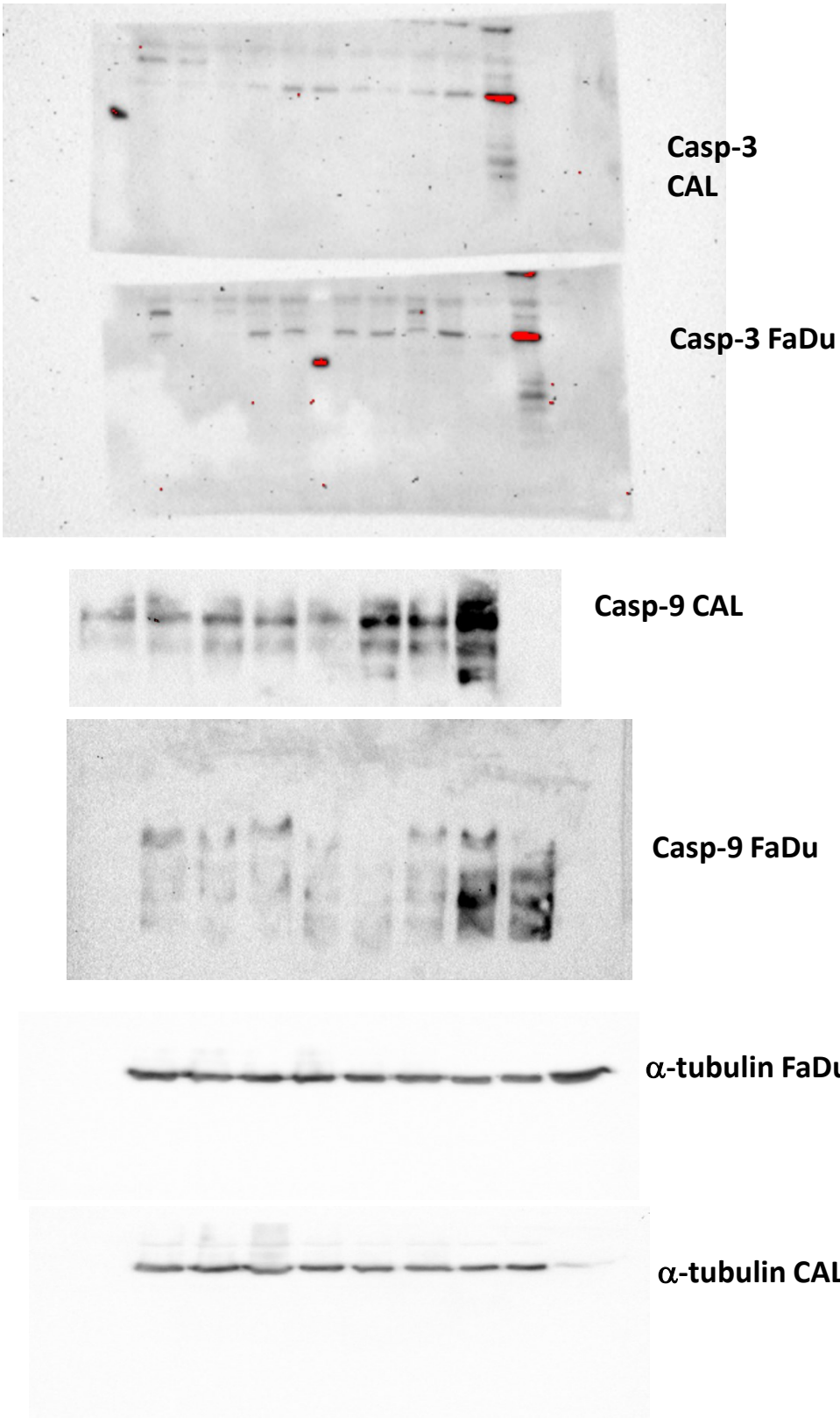

**Figure S2**

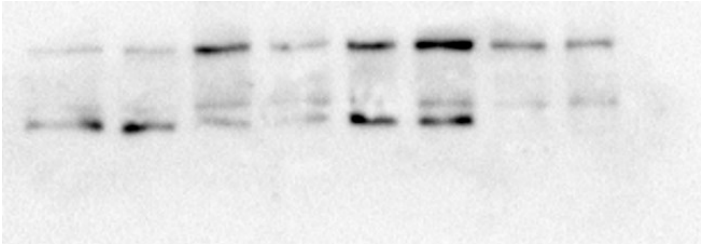

**HMGB1 FaDu**

**LC3 FaDu**

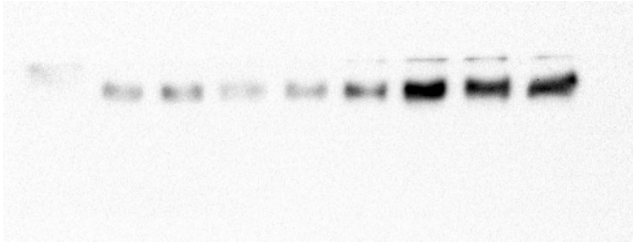

**HMGB1 CAL**

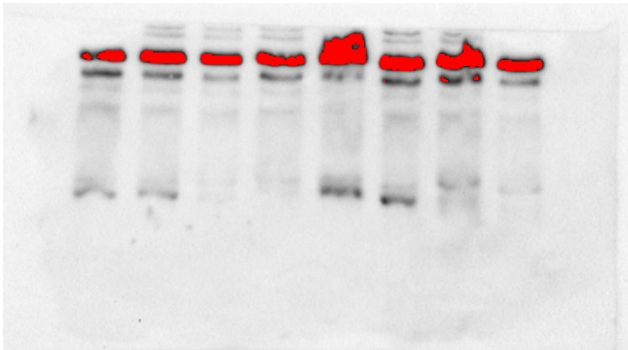

**LC3 CAL**

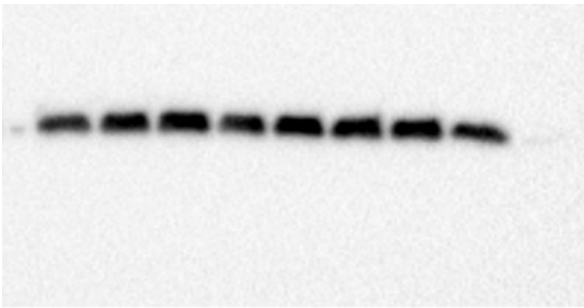

**GAPDH CAL**

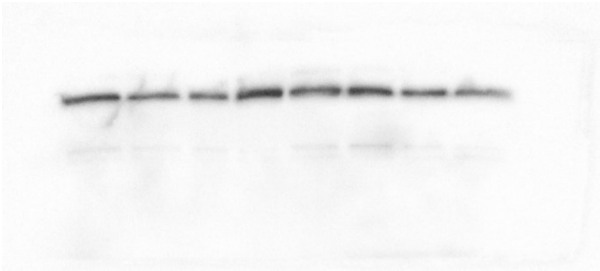

**GAPDH FaDu**

**Figure S3**

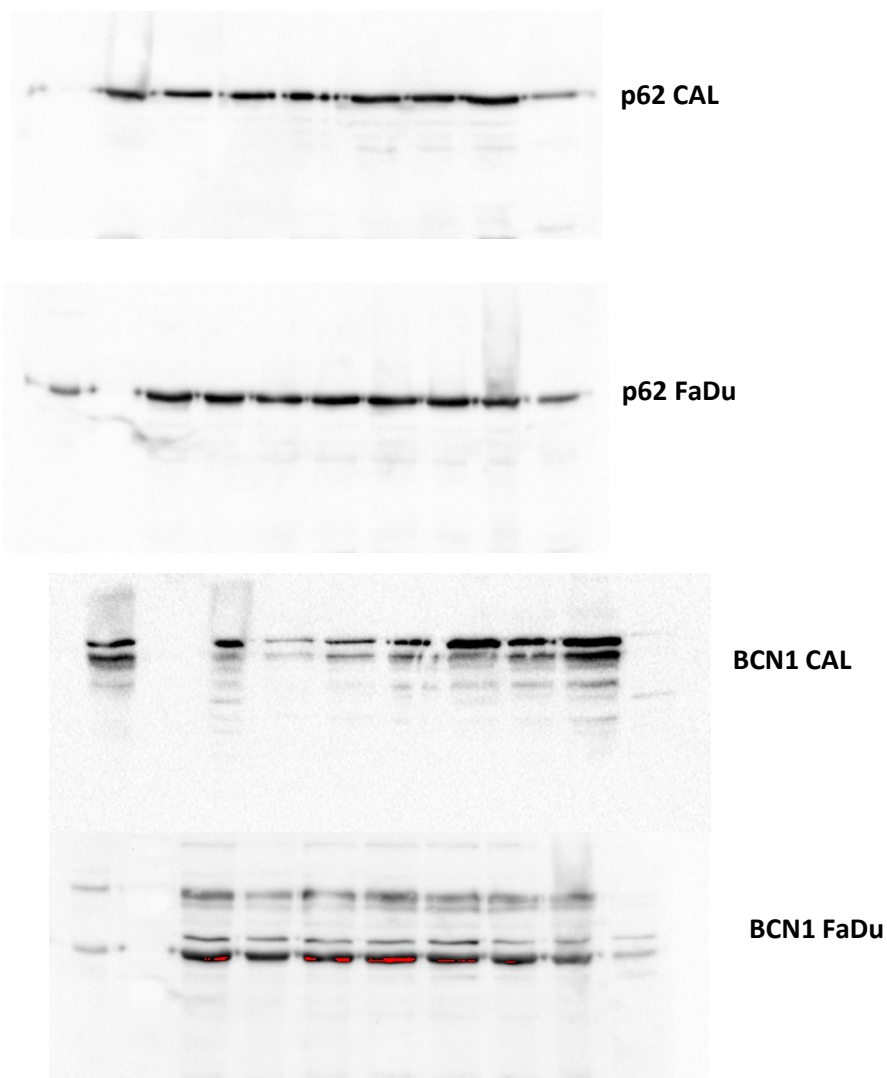

**Figure S4**

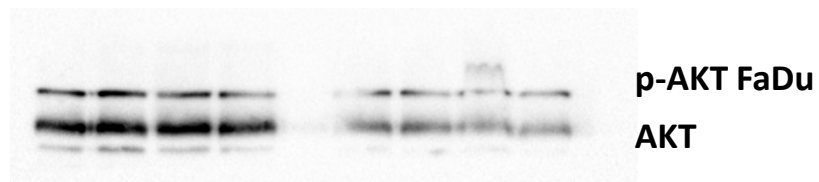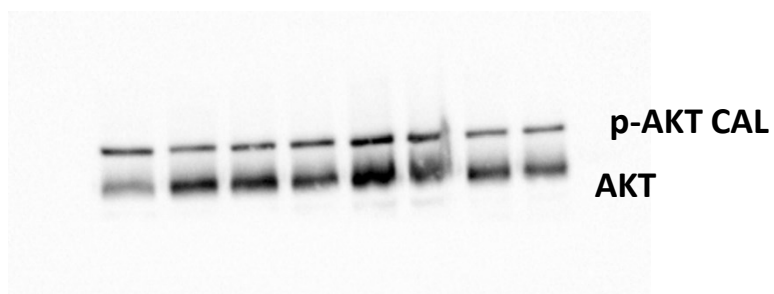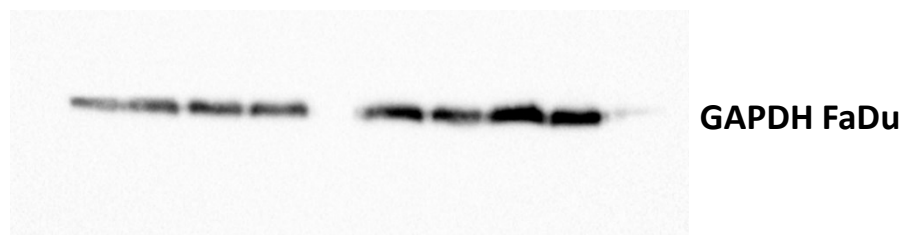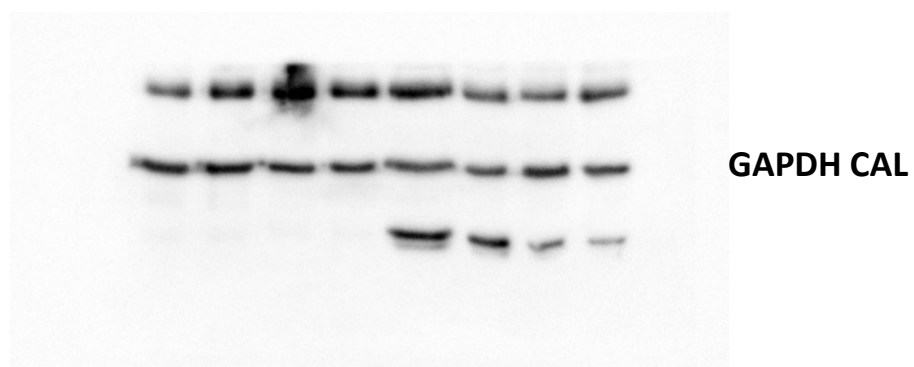

Supplement: Supplementary file 1 [file cancers-13-03867-s001.zip › cancers-1275383-supplementary.pdf]
